# Chinese Soft-Shelled Turtle Oil in Combination With Swimming Training Improves Spatial Memory and Sports Performance of Aging Rats

**DOI:** 10.3389/fphys.2021.660552

**Published:** 2021-05-28

**Authors:** Chia-En Yang, Tsung-Ming Yeh, Ching-Dong Chang, Wen-Ling Shih

**Affiliations:** ^1^Office of Physical Education, National Pingtung University of Science and Technology, Neipu, Taiwan; ^2^Department of Biological Science and Technology, National Pingtung University of Science and Technology, Neipu, Taiwan; ^3^General Research Service Center, National Pingtung University of Science and Technology, Neipu, Taiwan; ^4^Department of Veterinary Medicine, National Pingtung University of Science and Technology, Neipu, Taiwan

**Keywords:** soft-shelled turtle, ROS, DHEAS, swimming, aging

## Abstract

In this study, waste fat from the Chinese soft-shelled turtle (*Pelodiscus sinensis*) was used as the raw material, and soft-shelled turtle oil (SSTO) was extracted by water heating. Analysis of the fatty acid composition of SSTO revealed that unsaturated fatty acids (UFAs) comprised more than 70% of the oil, of which more than 20% were omega-3 poly-UFAs. DPPH radical scavenging and cellular ROS assays confirmed the reduction of oxidative stress by SSTO. In D-galactose-induced aging rats, SSTO feeding alone or in combination with swimming training resulted in improved memory and physical strength. In addition, SSTO feeding with swimming intervention significantly increased the SOD level and maintained better blood pressure in the aged rats. The serum DHEAS and soleus muscle glycogen level were also highly correlated with SSTO feeding and swimming training. In conclusion, the results of this study demonstrated that SSTO has the potential to be developed into a health food that exerts anti-aging effects, and those effects are stronger when combined with daily swimming exercise.

## Introduction

Aging is an irreversible phenomenon in which organisms undergo progressive and systemic complex physiological processes at the end of life, which are related to the complex interactions between genetics, acquired lifestyles, environment and chronic diseases. With improvement in quality of life, increasing health consciousness and the rapid advancement of medical science, people aged 65 years and older will comprise 16% of the population in 2050. A combination of internal and external factors causes aging, which is inevitable, but can be delayed; many aging-associated diseases are also avoidable (Siedner, 2019; Sabatini et al., 2021).

Oxidative damage affects many organs, and the brain is particularly vulnerable, as it consumes 20% of the body’s oxygen and contains large quantities of unsaturated fatty acids (UFAs) that are susceptible to oxidation (Barja, 2004). Studies have shown that oxidative stress causes neurodegenerative brain diseases, including Alzheimer’s and Parkinson’s diseases, which are directly related to free-radical damage (Song and Zhao, 2007; Calviello et al., 2013).

Increased free-radical reactions is known to promote the aging process, and inhibition of the accumulation of free radicals in cells can reduce the rate of aging and alter disease pathogenesis (Harman, 2003). Exercise is considered to be a beneficial factor in a healthy lifestyle, as physical activity can improve the antioxidant system and lower lipid peroxidation levels in the elderly (Bouzid et al., 2018). In addition to being able to delay aging, exercise is known to prevent cognitive decline; therefore, reducing antioxidative stress and increasing exercise levels are very important in old age. Sustained physical activity with antioxidant supplementation has been suggested to further shield the body from oxidative stress, which is especially beneficial in the elderly population (Simioni et al., 2018).

Chinese soft-shelled turtles (*Pelodiscus sinensis*) were popular in ancient times as a food and medicine, and are the most common farmed turtle in Asia. People in some areas of these countries still consume soft-shelled turtle meat (Yamanaka et al., 2016). Owing to people commonly believing that consumption of too much fat may lead to a high cholesterol level, along with turtle fat having a heavy fishy taste, it is normally discarded. Some companies have already developed health foods from soft-shelled turtle meat or eggs for use in humans and pets, but no scientific research has been conducted on the oil of the soft-shelled turtle in order to assess its health benefits.

Studies have shown that the benefits of swimming over other sports include reduced stress on bones and joints, a reduced risk of osteoporosis, improved cardiovascular health, maintenance of muscle mass and coordination, and more importantly, little to no injury, as no physical contact occurs (Jennings, 1997; Tanaka et al., 1997; Hart et al., 2001; Moreira et al., 2014). These factors make swimming especially suitable for the elderly. Therefore, in this study, we investigated whether rats fed with soft-shelled turtle oil (SSTO) had a better learning and memory performance in the Y-maze test and better physical endeavor in swimming training.

## Materials and Methods

### Preparation of Soft-Shelled Turtle Oil

Soft-shelled turtle fat obtained from Sanhe Biotech (Ligang, Pingtung, Taiwan) was used to prepare SSTO. Briefly, 100 g of fat were placed in a steel pan, stirred and heated to 100, 200, 300, or 400°C in water. An infrared thermometer was used to determine the temperature of the material. After the target temperature had been reached, the fat was continually stirred for an additional 10 min to obtain SSTO. After cooling, the oil was centrifuged at 10,000 × *g* and filtered through Advantec No. 1 filter paper to remove solid impurities or precipitation from the oil. The purified turtle oil was stored at 4°C.

### Cytotoxicity Assessment and Cellular Reactive Oxygen Species (ROS) Assay

Cytotoxicity analysis was performed using RAW264.7 cells (obtained from the Food Industry Development Research Institute) cultured in Dulbecco’s Modified Eagle’s Medium (DMEM) supplemented with 4 mM L-glutamine, 4.5 g/L glucose, 1.5 g/L sodium bicarbonate and 10% fetal calf serum (FCS). Analysis was carried out on cells treated with various concentrations of emulsified SSTO (1, 5 and 10%) or vehicle control (0.04% Tween-20) for 24 h, and the cell viability was determined using a 3-(4,5-dimethylthiazol-2-yl)-2,5-diphenyl tetrazolium bromide (MTT) cell viability and cytotoxicity assay.

In the cellular ROS assay, RAW264.7 cells were treated with various concentrations of SSTO for 2 h. Cells were then incubated with 2′,7′-dichloro-dihydrofluorescein diacetate (H2DCFDA) for 30 min, followed by treatment with H_2_O_2_ to induce ROS formation. The fluorescence intensities were determined as described in previous studies (Shih et al., 2018).

### DPPH Radical Scavenging Assay

Different concentrations of SSTO or vehicle control were compared with butylated hydroxytoluene (BHT) as the positive control. In this assay, 0.1 mM DPPH in methanol was prepared, and 2.4 mL of this solution were mixed with 1.6 mL of SSTO in methanol. The reaction mixture was vortexed thoroughly and incubated for 30 min in the dark. The absorbance of the mixture was measured spectrophotometrically at 517 nm. The resting DPPH level was calculated as the percentage of absorbance using the following formula: % DPPH level = 1-(OD_*control*_ – OD_*sample*_)/OD_*control*_ × 100%

### Animal Experimental Protocol

**FIGURE 1:**
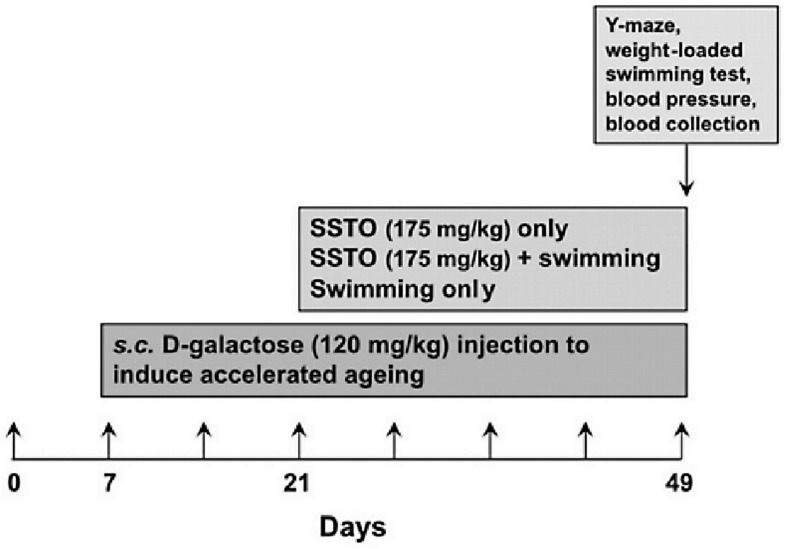
Flowchart of the animal experiment. One week after the animals had been allowed to adapt to the environment, the rats were injected subcutaneously with D-galactose to induce accelerated aging for 42 days. SSTO feeding and/or swimming training were started on day 14 after the D-galactose injections began and continued for 4 weeks. The outcomes were assessed using the Y-maze for learning memory performance and weight-loaded swimming for physical fitness testing. Blood pressure was also recorded and blood samples were collected for further analysis.

The animal study was approved by the Experimental Animal Care and Use Committee of National Pingtung National University of Science and Technology (Project number NPUST-109-041). Forty male 8-week-old Sprague-Dawley (SD) rats were purchased from BioLESCO Taiwan Co. The animals were housed in individual ventilated cages (IVCs) under 25 ± 1°C and a 12:12-h light:dark cycle. The experiment was initiated 1 week after the animals had been allowed to adapt to their new environment. The rats were divided into five groups (*n* = 8 for each group) as follows: Group 1, no treatment; Group 2, subcutaneous (s.c.) D-galactose (120 mg/kg) injection to accelerate aging; Group 3, D-galactose-induced aging and SSTO feeding alone; Group 4, D-galactose-induced aging and SSTO feeding plus swimming training; Group 5, D-galactose-induced aging with swimming training only. D-galactose was injected for 42 days, and SSTO feeding and exercise training started on day 14 after the injections began and lasted for 28 days in total. The amount of SSTO used was based on the recommendation for fish oil daily consumption of the Food and Drug Administration, Ministry of Health and Welfare, Taiwan, which is 2 g/day for humans (Taiwan Food and Drug Administration, 2020). According to a drug usage conversion coefficient between humans and rats (US Foof & Drug Administration, 2005), this is equal to 175 mg/kg/day for rats (assume adult human body weight is 70 kg, and the conversion factor for human equivalent doses to rat dose is known as 6.2; the dosage calculation formula: (2 g/70 kg) × 6.2 = ∼175 mg/kg). The complete procedure of the animal experiment was as shown in Figure 1. SSTO was fed to the animals by gavage at 10 a.m. daily, and the animals were allowed to freely consume food.

### Y-Maze Test

Rodents have the habit of exploring new environments. The Y-maze consists of three arms, labeled A, B, and C. At the start of the test, the C arm was closed off, and the animal was allowed to explore the start of arms A and B for 5 min, then placed back into the home cage and allowed to rest for 3 min. The maze was wiped with alcohol to remove any smell. Then, all three ends of the maze arms were opened, and the rat was allowed to explore freely for 10 min, during which the number of times and the times at which the rat entered the three arms, in addition to the speed of movement, were recorded (Dellu et al., 2000). Spontaneous alteration behavior was defined as (consecutive entries into three different arms/total number of arm entries – 2) ×100; a higher percentage showed improved memory and cognition (Muhammad et al., 2019).

Then, in order to evaluate the effects of SSTO and swimming on short-term spatial memory, with all three arms open, a food reward was placed at the end of arm C, and the animal was placed into arm A. After three sessions of training to enable the animal to recognize that the food was always placed in arm C, the animal was placed back in the home cage. After 1 day of rest and 8 h of starvation, the spatial memory of the animal was then tested in terms of its ability to remember that the food was located in arm C. The search for food was conducted 5 times, within a duration of 10 min. A digital camera was positioned above and software employed for analysis as described in the previous section.

### Regular Swimming Training and Weight-Loaded Forced Swimming

A water tank was employed for swimming training, of a diameter of 54 cm and a height of 65 cm. The water temperature was maintained at 34 ± 1°C. Training began at 2 weeks after the start of accelerated aging induction (Figure 1) and was conducted at 11 AM every day. During the training, the rats voluntarily swam free of load, and the daily swimming duration was 10 min. After training, the rats were gently towel-dried and warm air-dried before being returned to their cages. Weight-loaded forced swimming was performed until exhaustion with a weight of 5% of the animal’s body weight attached to the tail (da Silva et al., 2016; Lin et al., 2018). Animals were assessed as being fatigued when they failed to rise to the surface of the water to breathe within an 8-s period. The rats were gently towel-dried and warm air-dried immediately after the exercise.

### Blood Pressure Measurement and Blood Sample Collection

The rats were placed in plastic restrainers, and a cuff with a pneumatic pulse sensor was attached to the tail and fixed with surgical tape. Blood pressure was recorded on a non-invasive rodent blood pressure monitor (MK-2000ST, Muromachi Kikai, Japan). Blood samples were collected from the tail vein and centrifuged at 1,000 × g for 10 min to separate the serum.

### Biochemical Analysis of Serum

Commercial superoxide dismutase (SOD) and total antioxidant capacity (TAC) assay kits were used to measure the SOD and TAC levels in serum samples of the rats. The processes were as described in the instructions of the manufacturers. A dehydroepiandrosterone sulfate (DHEAS) ELISA kit (Wuhan Fine Biotech Co., Inc.) was used to measure the level of DHEAS.

### Glycogen Level in the Soleus Muscle

The glycogen levels in soleus muscle samples were measured using a glycogen colorimetric assay kit (BioVision) according to the manufacturer’s instructions. Soleus was selected as the target muscle for this study as studies have shown that soleus muscle is one the key muscle types that can be improved with swimming training in rat model (Forte et al., 2020; Moustafa and Arisha, 2020).

### Statistical Analysis

The results of this study were analyzed using Excel 2010. The unpaired *t*-test was employed for statistical analysis to compare data from treated groups with those of the aged rats with saline treatment, and to compare data from the SSTO or swimming group with those of the SSTO+swimming group, using Prism software (v.6.0; GraphPad Software, San Diego, CA, United States). A *p*-value of <0.05 was considered as significant. Data are presented as mean ± SD.

## Results

### SSTO Yield and Fatty Acid Analysis

The yields of SSTO extracted at different rendering temperatures indicated that the yield increased with increasing rendering temperature, being 30% at 100°C and 65% at 400°C. Fatty acid analysis was conducted by the Center for Agricultural and Aquacultural Products Inspection and Certification at NPUST. Table 1 lists all the types of fatty acid that accounted for more than 1% of the total fatty acid content. The results showed that the SSTO extracted at 100°C, similar to fish oil, had high levels of UFAs, at approximately 70%, including 22% omega-3 poly-UFAs and 29.78% omega-9 mono-UFAs.

**TABLE 1:** Fatty acid composition of the SSTO.

	Types of fatty acid	Content (%)
omega-9 mono-UFAs	Oleic acid	27.98
omega-7 mono-UFAs	Palmitoleic acid	6.52
omega-6 poly-UFAs	Linoleic acid	8.50
	Arachidonic acid	2.12
omega-3 poly-UFAs	Docosahexaenoic acid (DHA)	12.97
	Eicosapentaenoic acid (EPA)	5.37
	Docosapentaenoic acid (DPA)	2.62
	Alpha-linoleic acid	1.04
SFAs	Palmitic acid	20.27
	Steric acid	5.94
	Myristic acid	3.55
	Total	96.88

### Assessment of Free-Radical Scavenging Activity of SSTO *in vitro* and in Cultured Cells

**FIGURE 2:**
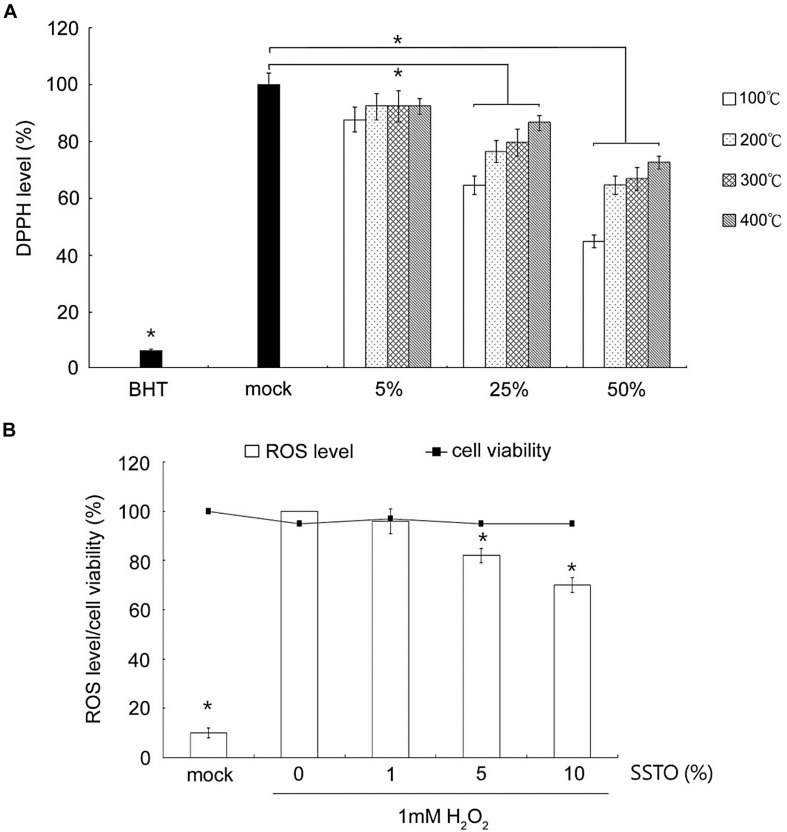
Antioxidant activity of SSTO. **(A)** DPPH assay. The *y*-axis shows the relative percentage of the residual DPPH level as compared with butylated hydroxytoluene (BHT) as the positive control. The *x*-axis shows the positive and negative controls and SSTO samples at different concentrations prepared at various rendering temperatures. **(B)** Cellular ROS assay. RAW264.7 cells were incubated with SSTO, and the percentage of ROS reduction in the culture was measured. Cell viability was also monitored by MTT assay. *Statistically significant difference as compared with the non-SSTO treated group, but with 1 mM H_2_O_2_.

Our results showed that SSTO at concentrations of 25 and 50% had a significantly high antioxidant activity. SSTO prepared at lower rendering temperatures had a better free radical scavenging effect than samples prepared at higher rendering temperatures (Figure 2A). In order to measure the ability of SSTO to reduce oxidative stress in cells caused by H_2_O_2_, an ROS assay using non-cytotoxic concentrations of SSTO was performed. The results showed that SSTO (extracted at 100°C) at 5 and 10% significantly reduced oxidative stress and had a high ROS scavenging activity (Figure 2B). As SSTO prepared under 100°C has the best antioxidant capacity, all subsequent experiments utilized SSTO rendered at that temperature.

### Assessment of Aging in the Rats Treated With SSTO

**FIGURE 3:**
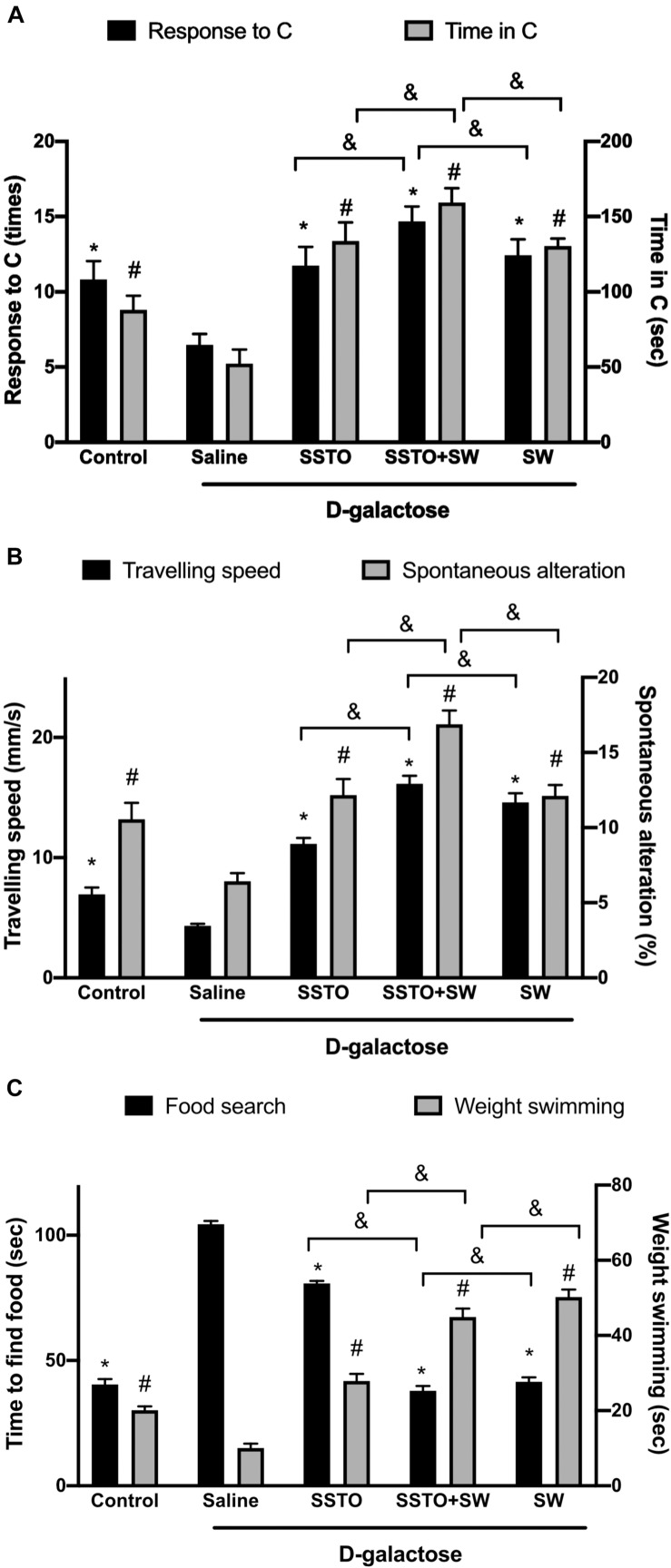
Effects of SSTO and swimming training on aging rats. **(A)** Exploration evaluation. The left *y*-axis (black bars) shows the number of times that the rats entered the new environment (arm C) within 10 min, and the right *y*-axis (white bars) shows the average duration for which the animals stayed in arm C. **(B)** Mobility determination. The left *y*-axis (black bars) shows the traveling speed, and the right y-axis (white bars) indicates the spatial learning ability, as evaluated by the spontaneous alteration percentage. **(C)** SSTO and swimming training improved short-term spatial memory (black bars) and physical performance (white bars). Left *y*-axis: average time to find food; right *y*-axis: endurance measured as weight-loaded forced swimming duration. *^#^Statistically significant difference as compared with the aged rats with saline treatment; ^&^statistically significant difference when compared with SSTO + swimming group.

As shown in Figures 3A,B, compared with the control rats, D-galactose-induced aging rats lost the desire to explore new environments, and exhibited poor mobility and spatial learning. The aging rats fed with SSTO alone, those with SSTO feeding in combination with swimming training, and those with swimming training alone all exhibited greater exploration of a new environment and longer stays in arm C in the Y-maze test, in addition to a greater speed of movement and a higher percentage of direction alteration behavior. Among the groups, the rats with SSTO feeding in combination with swimming training exhibited the best anti-aging response. These results indicated that SSTO together with swimming training had the best outcome in terms of improving cognition, which led to enhancement of the motivation and desire of the aging rats to explore new environments.

Further, using the Y-maze with food as a reward, the short-term spatial memory and sports performance in the different groups of rats were compared. As shown in Figure 3C (left *y*-axis), the aging rats required a greater amount of time to find the food than the normal rats, indicating that D-galactose injection caused deterioration in cognitive memory. All the rats in the treatment groups with SSTO feeding and/or swimming training exhibited improvement in short-term spatial memory, especially the rats with SSTO feeding in combination with swimming training, in which the results were most similar to those of the normal rats. As shown in Figure 3C (right *y*-axis), the aging rats were less physically fit than the normal rats, and the duration of weight-loaded swimming was reduced. When the aging rats were fed with SSTO and/or completed swimming training, their swimming performance was improved. Combined SSTO feeding and swimming training improved the duration of weight-loaded swimming to a level that was four times that of the aged rats with no treatment or training.

### Effects of SSTO and Swimming Training on the Regulation of Blood Pressure

In normal rats, the systolic blood pressure (SBP) ranges from 84–134 mmHg, and the diastolic blood pressure (DBP) is around 60 mmHg. In our study, the D-galactose-induced aging rats without treatment or intervention had significantly higher SBP and DBP values than the control group. Aged rats with SSTO feeding and/or swimming training all showed significant improvement in terms of a lowered blood pressure. In particular, in rats with SSTO feeding in combination with swimming training, the SBP and DBP values were lower, to near the ranges of normal rats (Table 2). Therefore, SSTO has the potential to be developed into a health food product for blood pressure control.

**TABLE 2:** Systolic and diastolic blood pressure and mean blood pressure of the rats in this study.

	Blood pressure		
	
Group	SBP (mmHg)	DBP (mmHg)	Average (mmHg)
Control	116.0 ± 15.4*	63.3 ± 21.5*	81.3 ± 24.6*
Aged rats	152.3 ± 10.5	115.8 ± 19.0	128.0 ± 15.2
Aged rats + SSTO	141.8 ± 20.1	73.2 ± 12.2*	96.0 ± 14.1*
Aged rats + SSTO + swimming	109.3 ± 35.3*	74.5 ± 23.2*	86.0 ± 25.8*
Aged rats + swimming	140.3 ± 27.3	78.0 ± 8.4*	98.8 ± 12.5*

*Statistically significant differences as compared with the aged rats without treatment. Data presented as mean ± SD. SBP, systolic blood pressure; DBP, diastolic blood pressure.

### Serum Antioxidant Enzyme Activity and DHEAS Analysis

**FIGURE 4:**
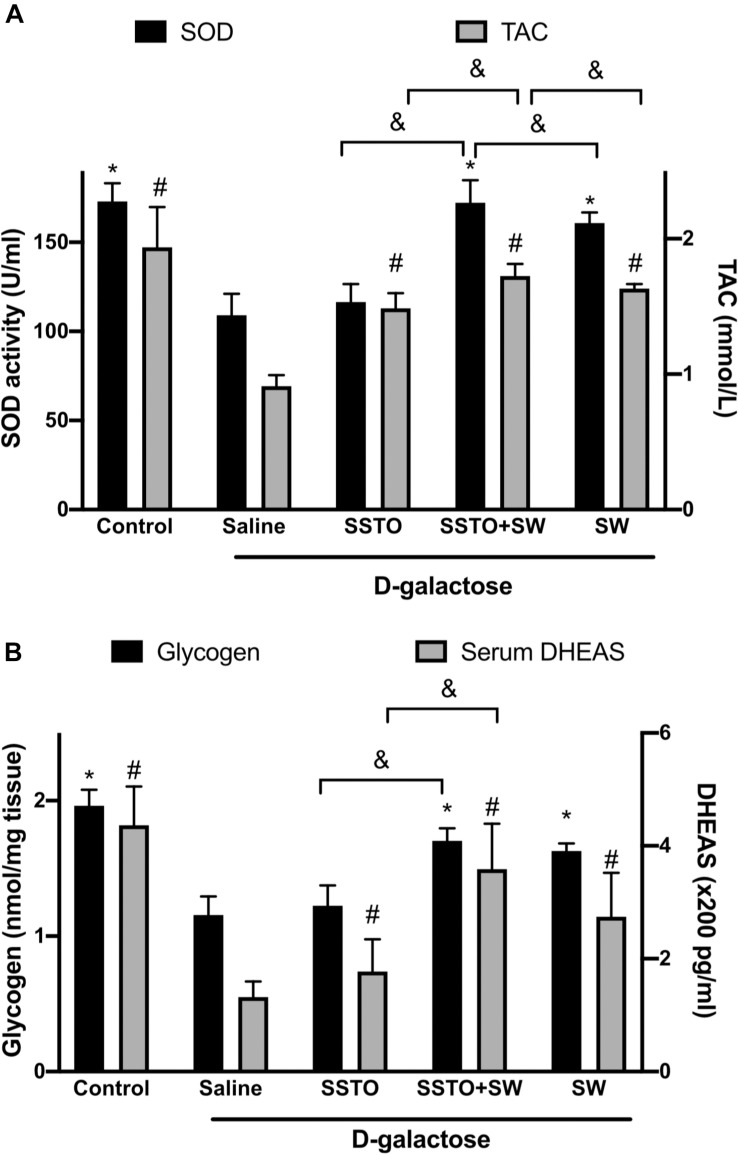
Biochemical analysis of serum and muscle. **(A)** Effects of SSTO and/or swimming on serum SOD (black bars) and TAC (white bars) antioxidant enzyme activity. **(B)** Glycogen content (black bars; left *y*-axis) in the soleus muscle and serum DHEAS level (white bars; right *y*-axis). *^#^Statistically significant difference as compared with the aged rats with saline treatment; ^&^statistically significant difference when compared with SSTO + swimming group.

The antioxidant enzyme activities of SOD and TAC in the serum were compared between groups. As shown in Figure 4A, the rats with SSTO feeding alone did not have an increased serum SOD level as compared with the aging rats, while rats with swimming training alone or SSTO feeding in combination with swimming had significantly higher SOD levels. Additionally, SSTO feeding in combination with swimming resulted in the best outcome with regards to the TAC level in the aged rats. DHEAS is a metabolite of dehydroepiandrosterone (DHEA) that circulates in the blood, and measurement of DHEAS in the blood is a reliable approach by which to show the bioactive level of DHEA. DHEA is known to play important roles in neurological functions, and has been suggested to be associated with the development of neurodegenerative diseases in humans. As shown in Figure 4B (right *y*-axis), rats with D-galactose-induced aging that did not receive treatment exhibited a significantly decreased DHEAS level, while swimming training with or without SSTO feeding resulted in increased DHEAS levels in the aged rats. Aged rats with SSTO feeding alone did not exhibit this effect.

### Glycogen Level in the Soleus Muscle

Kinesiology study has shown that the soleus muscle is the primary muscle used in exercise in rats. This muscle is highly-correlated with exercise endurance, and therefore, strengthening of the soleus muscle with regular excise will improve physical performance and can decrease soreness, pain and spasm after exercise. The glycogen levels in the soleus muscle in the different groups were compared. As shown in Figure 4B (left *y*-axis), in the aging rats, the soleus muscle glycogen was significantly depleted, while the glycogen levels of the rats with swimming alone or swimming combined with SSTO feeding were significantly increased, suggesting that exercise is important in order to preserve a high level of glycogen in the muscles. SSTO feeding alone did not boost the glycogen level in the aged rats.

## Discussion

In this study, Chinese SSTO was extracted from waste fat after turtle slaughter, and the fat was transformed into a useful bioactive agent and a valuable aquacultural by-product. However, the strong fishy smell will need to be eliminated when it is developed as a product for human consumption. The results of the present study showed that a higher rendering temperature resulted in a higher oil yield, but a decreased antioxidant activity. A previous study compared the extraction of SSTO by water bath, frying, ethyl acetate extraction and n-hexane extraction, then measured the changes in the acid value, peroxide value and fatty acids after storing the oil samples for 60 days. The results showed that the yield of extraction by chemical solvents was significantly higher than that of traditional physical methods, and the oil obtained by high-temperature rendering turned rancid more quickly (Lee, 2017). Our present study also showed that the antioxidant activity of SSTO extracted at higher rendering temperatures was poor, suggesting that a high rendering temperature may damage the fatty acids and other bioactive components.

The prevention and treatment of dementia has long been a major topic in research. A study performed previously, in which participants were followed-up for more than 20 years, showed that saturated fatty acid (SFA) intake is associated with dementia and Alzheimer’s disease, while UFAs have a protective effect (Laitinen et al., 2006). Another long-term study showed that replacing 5% of the total calories with poly-UFAs and mono-UFAs reduced the risk of cardiovascular disease (Hu et al., 1997). The results of our study showed that UFAs comprise more than 70% of SSTO. Although this percentage is lower than that of vegetable oils, such as olive oil and bitter tea oil, a UFA content of 70% shows it to be a high-quality oil among animal oils, especially as it contains high levels of omega-3, -6 and -9, and eicosapentaenoic acid (EPA) and docosahexaenoic acid (DHA) at 5.37 and 12.97%, respectively. As EPA and DHA have been shown to effectively reduce triglycerides (Klingel et al., 2019), our results suggested that SSTO is a good healthy oil.

Omega-3 fatty acids present in fish oil have been shown to be of therapeutic potential with regards to several neurodegenerative and neurological disorders (Dyall, 2015), and EPA and DHA have been demonstrated to have important roles in maintaining neuronal cell functions (Reimers and Ljung, 2019). Based on information from the US Department of Agriculture, per 100 g of fish oil, the contents of EPA, DPA and DHA are 6.898 g, 0.935 g and 10.968 g, respectively (Agriculture UDo, 2019). Our results showed that SSTO has higher DHA (12.97 g) and DPA levels (2.62 g), suggesting that EPA and DHA may be key to the anti-aging effects of SSTO. Additionally, SSTO has a high content of oleic acid (at 27.98%), an omega-9 fatty acid that has been demonstrated to lower the level of low-density lipoprotein (LDL) cholesterol and prevent arteriosclerosis. This further indicates that SSTO has a great potential to be developed as a health food supplement. Based on the results of our study and those reported in the literature, SSTO possesses the beneficial characteristics of both vegetable oil and high-quality animal oil, which render it suitable for development as an anti-aging supplement, SSTO being rich in omega-3 UFAs, which are most often lacking in the diet.

In studies of yeast and fruit flies, and in mouse models, it was found that superoxides may damage the genomic integrity and stability, contributing to acceleration of aging and shortening the lifespan (Villani et al., 2013). Antioxidant supplements are therefore thought to remove free radicals and boost the enzyme system within the body, which disarms free radicals and helps to delay aging. The most important antioxidant enzyme system in the body is located in the cytosol and mitochondrial intermembrane space. The system utilizes SODs to convert superoxide (O2^–^) into two less-damaging species, H_2_O_2_ and oxygen. In the present study, we found that SSTO directly scavenged superoxide radicals from cultured cells and increased the endogenous SOD enzyme activity in an aging rat model, and its antioxidant effect was greater when combined with regular swimming training. Further studies are needed to determine whether the antioxidant effect of SSTO is mediated by regulation of cell signaling pathways, such as mitogen-activated protein kinases (MAPKs), antioxidant/electrophile response element (ARE/EpRE) and transcription factor Nrf2, in the host. Whether other antioxidant enzymes in the body, such as catalase (CAT) in peroxisomes and glutathione peroxidase (GP), which may scavenge both ROOH and H_2_O_2_ (Vecchione et al., 2007), are also enhanced after SSTO administration is also an area most worthy of investigation.

Many studies have shown that exercise has positive effects on cognitive and other brain functions (Cotman and Berchtold, 2002; Cotman et al., 2007). In studies of rodents, voluntary wheel-running was shown to increase the gene expressions of neurotrophic factors and other neuroplasticity-related genes in the hippocampus, such as calmodulin-dependent protein kinase II, MAPK, and CREB (Neeper et al., 1996; Tong et al., 2001). Cellular factors that mediate the anti-aging effects of SSTO need to be elucidated.

The soleus muscle is one of the triceps surae muscles located at the calf. It starts at the upper portions of the fibula and tibia, and plays a major role in plantar flexion. It has more red muscle fibers than the gastrocnemius, hence it is also known as the “endurance muscle.” Studies have shown that people with better endurance have lower mortality rates, and this same trend exists across all age groups (Montori-Grau et al., 2009). The main determinant of muscle endurance is glycogen, the main energy substrate that can produce anaerobic energy by releasing glucose to be used by the muscle fibers. The results of the present study demonstrated that SSTO feeding in combination with swimming training had a positive effect on the glycogen level in the soleus muscle.

DHEA and DHEAS are secreted by the adrenal glands. They are known to be multi-functional hormones that are associated with immunity, diabetes, growth of neurons, memory and cognition, and aging. A long-term follow-up study of 27 years’ duration showed that the DHEAS level in the serum can predict longevity in men (Enomoto et al., 2008). So far, it is not clear what factors are linked to the serum DHEAS level, but it is relatively certain that an increase in the serum DHEAS concentration may extend the life span. The present study also confirmed that SSTO feeding and exercise attenuated DHEAS level decrease caused by D-galactose induced aging, although the level in the aged rats with SSTO feeding and swimming was still lower than that in normal young rats (Figure 4B).

Although forced exercise such as treadmill and swimming training, or voluntary roller exercise, are commonly used in experimental settings to train rodents, whether or not forced and voluntary exercise differentially affect brain functions is not yet known. However, the maze test is the most acceptable method by which to study cognition and memory in experimental animals. In this study, we used a simple Y-maze to study the effects of SSTO feeding and swimming in an aging rat model. In the future, we plan to use more advanced behavioral analysis methods to explore the mechanisms of SSTO involved in slowing the aging process, improving brain function, increasing physical fitness, and even prolonging lifespan in animal models.

## Data Availability Statement

The raw data supporting the conclusions of this article will be made available by the authors, without undue reservation.

## Ethics Statement

The animal study was reviewed and approved by the IACUC of National Pingtung University of Science and Technology.

## Author Contributions

C-EY and T-MY performed the cell culture-based analyses and animal tests, and drafted the manuscript. C-DC participated in the design and coordination of the study and helped to draft the manuscript. W-LS, the principal investigator of the study were responsible for the grant application, interpretation of the results, and manuscript editing. All authors read, commented on, and approved the final manuscript.

## Conflict of Interest

The authors declare that the research was conducted in the absence of any commercial or financial relationships that could be construed as a potential conflict of interest.
